# Speculation on optimal numbers of examined lymph node for early-stage epithelial ovarian cancer from the perspective of stage migration

**DOI:** 10.3389/fonc.2023.1265631

**Published:** 2023-09-22

**Authors:** Yuan Li, Jiashan Ding, Huimin Zheng, Lijiang Xu, Weiru Li, Minshan Zhu, Xiaolu Zhang, Cong Ma, Fangying Zhang, Peiwen Zhong, Dong Liang, Yubin Han, Siyou Zhang, Linsheng He, Jiaqi Li

**Affiliations:** ^1^ Department of Obstetrics and Gynecology, First People’s Hospital of Foshan, Foshan, Guangdong, China; ^2^ Department of Gynecological Oncology, Xiangya Hospital Central South University, Central South University, Changsha, Hunan, China; ^3^ Department of Gynecologic Oncology, Jiangxi Maternal and Child Health Hospital, Nanchang Medical College, Nanchang, Jiangxi, China

**Keywords:** stage migration, structural breakpoint, early-stage, epithelial ovarian cancer, examined lymph node, survival

## Abstract

**Introduction:**

In early-stage epithelial ovarian cancer (EOC), how to perform lymphadenectomy to avoid stage migration and achieve reliable targeted excision has not been explored in depth. This study comprehensively considered the stage migration and survival to determine appropriate numbers of examined lymph node (ELN) for early-stage EOC and high-grade serous ovarian cancer (HGSOC).

**Methods:**

From the Surveillance, Epidemiology, and End Results database, we obtained 10372 EOC cases with stage T1M0 and ELN ≥ 2, including 2849 HGSOC cases. Generalized linear models with multivariable adjustment were used to analyze associations between ELN numbers and lymph node stage migration, survival and positive lymph node (PLN). LOESS regression characterized dynamic trends of above associations followed by Chow test to determine structural breakpoints of ELN numbers. Survival curves were plotted using Kaplan-Meier method.

**Results:**

More ELNs were associated with more node-positive diseases, more PLNs and better prognosis. ELN structural breakpoints were different in subgroups of early-stage EOC, which for node stage migration or PLN were more than those for improving outcomes. The meaning of ELN structural breakpoint varied with its location and the morphology of LOESS curve. To avoid stage migration, the optimal ELN for early-stage EOC was 29 and the minimal ELN for HGSOC was 24. For better survival, appropriate ELN number were 13 and 8 respectively. More ELNs explained better prognosis only at a certain range.

**Discussion:**

Neither too many nor too few numbers of ELN were ideal for early-stage EOC and HGSOC. Excision with appropriate numbers of lymph node draining the affected ovary may be more reasonable than traditional sentinel lymph node resection and systematic lymphadenectomy.

## 1 Introduction

Epithelial ovarian cancer (EOC) is one of the most common gynecological malignancies with the 5-year survival rate of only 49.1% in advanced patients (1). Benefiting from comprehensive surgical staging and optimal (residual lesions < 1 cm) debulking surgery, which includes hysterectomy, bilateral salpingo-oophorectomy and lymph node dissection, the survival rate of early-stage EOC ranges from 70% ~ 95% (1, 2). Thus, surgery is a cornerstone in the treatment of early-stage EOC. When it comes to the lymphadenectomy, the strategy, scope and extent of lymph node dissection in the early-stage EOC are still uncertain (3–7).

Theoretically, a first and foremost purpose of lymph node dissection in the early-stage EOC is to obtain enough lymph nodes for pathological evaluation to accurately stage patients, predict future disease progression and guide management. However, considering that tumor-infiltrating lymph nodes often do not have visible changes in appearance, early-stage EOC with occult lymph node metastases tends to have too few resected lymph nodes to find the positive one. Thus, these advanced disease, which may have a worse prognosis than early-stage EOC and a better prognosis than other advanced patients, have been assigned to early stage in fact and become the main population for lymph node stage migration frequently (8). When stage migration occurs in early-stage patients with occult lymph node metastases, the prognostic assessment of three different patient groups will be inaccurate. Early-stage EOC will have decreased survival due to the presence of such understaged patients. Advanced EOC will also have decreased survival due to the exclusion of such advanced patients. For the understaged patient itself, not only the survival will be overestimated, but also the adjuvant treatment recommended after surgery will be inadequate owing to stage migration.

Hence, the spatial extent and number of examined lymph node (ELN) should be determined for early-stage EOC to accurately stage patients and to avoid the occurrence of stage migration. The spatial distribution of metastatic lymph nodes depends on the arrangement of the lymph network responsible for the drainage of the affected ovary, thus, the targeted compartmental lymphadenectomy (4–7), including removal of the malignancy together with its draining lymphatic network, may be a suitable strategy and method for determining the spatial extent of lymphadenectomy. However, as for the number of ELN, it was inconsistent in different prospective studies (9–11), and definitions of “enough lymph nodes removed” were also vague in retrospective studies with proposed numbers of ELN varying greatly from 1, 8, 10, 12, to 22 (12–17). These studies determined the cut-off number of ELN based on subjective wills or based on methods that were not multivariable or not statistically robust for early-stage EOC, or even applied the recommended ELN number for other cancers to EOC. More importantly, all of them have been limited to describing the improved prognosis associated with ELN numbers (9, 12–16, 18–20), and none have directly addressed the notion of stage migration (8, 21, 22).

To tackle these great controversies between guidelines and studies, we herein investigated the Surveillance, Epidemiology, and End Results (SEER) program to provide a more robust conclusion on the association of ELN number with staging and survival in early-stage EOC (23). By modeling, fitting and chow test, we proposed well-founded threshold numbers of ELN, which aimed at not only giving early-stage EOC a better prognosis, but also detecting occult or potential positive lymph node to avoid node stage migration and to provide a reference in the number of ELNs for reliable targeted compartmental lymphadenectomy.

## 2 Materials and methods

### 2.1 Data source and processing

The clinical data of EOC from 1992 to 2015 in the SEER database was obtained through the SEERStat port (23). Only stage T1M0 patients with ELN ≥ 2 were eligible. Screening was performed as follows: 1. Cases with incomplete or discrepant data on age, lymph node dissection, ELN, positive lymph node (PLN), TNM stage, year of diagnosis, primary site, laterality, histopathology, differentiation and follow-up data were removed; 2. Given that preoperative needle biopsy or intraoperative nodal sampling was common during the diagnosis and treatment of EOC, cases with ELN 0 and 1 were removed; 3. This study focused on the issue of lymph node dissection for early-stage EOC, so all patients with stage T2, T3 and M1 were excluded. The histopathological subtype of SEER data is annotated with ICD-O morphology/behavior codes. However, this annotation method is different from the actual clinical pathological classification of EOC. We referred to the research of Jennifer A. Doherty to correct histopathological subtypes (24), which includes serous, endometrioid, clear cell, mucinous, carcinosarcoma, Brenner & not otherwise specified (NOS) and mixed type. Endometrioid ovarian cancer with grade 3 & 4 were classified as high-grade serous ovarian cancer (HGSOC). Finally, a total of 10372 EOC cases were obtained for study, in which 2849 cases were HGSOC. See **Table S1** and **S2** for the specific number of patients, ELN and PLN for each subgroup.

### 2.2 Statistical analysis

Multivariate logistic regression, Cox regression and multiple linear regression models were used to analyze the effect of ELN number on the identification of lymph node stage migration, disease-specific survival (DSS) and PLN number. Lymph node metastasis status, PLN number and survival data of each sample were dependent (outcome) variables in these three models respectively. As a continuous or dichotomous variable, which ranged from 2 to 38, the number of ELN was an independent variable in different regression models. Several preoperative and intraoperative clinical information, such as age, TNM stage, laterality, primary site, differentiation and histopathological subtypes were included as controlled variables to correct the effect of ELN number. Postoperative clinical information was useless for determining the number of ELN during the surgical procedure, thus chemotherapy and secondary surgery and so on were not candidate variables for model building. In these three models, removal of 2 lymph nodes (ELN 2) was used as a reference. Odds ratios (ORs) for logistic regressions, coefficients (Coef) for multiple linear regressions, hazard ratios (HRs) for Cox regressions and 95% confidence intervals (CIs) of removal of 3~38 lymph nodes (ELN 3 ~ 38) were calculated respectively.

LOESS fit, a nonparametric procedure widely used for smoothing scatter plots to assess the relationship between continues variables, was used to find smooth curves based on ORs, exp (Coef) and HRs of different ELN numbers. A higher span smooths out the fit more, while a lower span captures more trends but introduces statistical noise if there is too little data. In this study, all fitting routines were performed using span 1.

Chow test was a common hypothesis testing method, which we used to determine the structural breakpoint of ELN number to avoid the occurrence of stage migration, to detect more PLNs and to improve outcomes of early-stage EOC patients. The basic idea was to divide a data set into two or more subsets on the grounds of the predictor variable and fit a regression model for each subset respectively. Then, based on the same sample data, a comparison between the mean regression coefficient of each subset across the changing of predictor variable was used to determine whether there was a significant difference between these two regression models, whether the fitted regression lines by LOESS had different shapes in these two subsets and whether the extent to which the predictor variable affected the outcome variable differed between these two subsets. According to the results of Chow test, if the p value was less than the minimal level of significance, the null hypothesis that fitted regression models were similar in these two subsets should be rejected. This meant that the effect of the predictor variable may differ in these two subsets. If the p value was not less than the minimal level of significance, the null hypothesis should not be rejected, indicating that there was no significant difference in these two regression models and the effect of the predictor variable may similar in these two subsets. In this study, different numbers of ELN were predictor variables in the Chow test and cut-off values for dividing a data set into subsets.

All of the above calculations and visualizations were done using R (version 4.0.1). Packages including survival, ggplot2, strucchange, rms, stringr, gridExtra, ggbreak and reshape2 were used with the default parameters.

## 3 Results

### 3.1 Patient characteristics and distributions of the ELN and PLN

**Figure 1:**
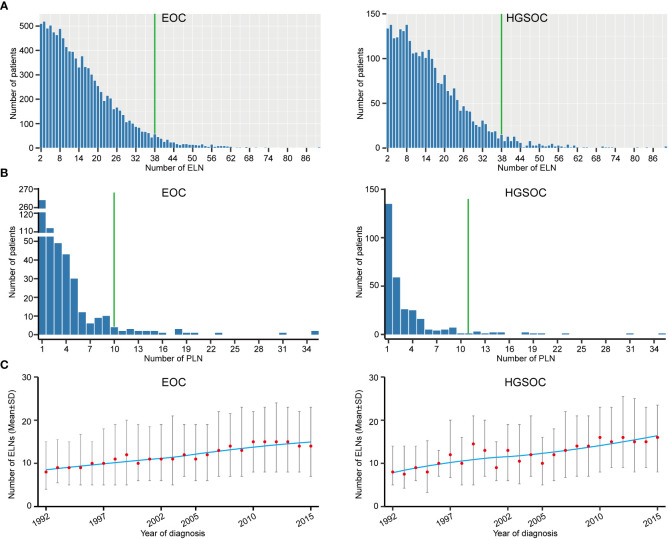
Distributions of examined lymph node (ELN) and positive lymph node (PLN). **(A)** Frequency distribution of ELN numbers for early-stage EOC and HGSOC. **(B)** Frequency distribution of PLN numbers for early-stage EOC and HGSOC. **(C)** Temporal trends of ELN number for early-stage EOC and HGSOC.

A total of 10372 eligible EOC patients with stage T1M0 and ELN ≥ 2 were analyzed. Reasons for exclusion were detailed in Method. Patient characteristics and distributions of the ELN and PLN were shown in **Table S1** and **Figure 1**. The median number of ELN and PLN was 13 and 2, respectively. Considering that 95% of patients had less than 38 ELNs and 95% of stage N1 patients had less than 10 PLNs (**Figures 1A, B**), subsequent analyses were limited to 2 ~ 38 ELNs. From 1992 to 2015, the ELN number of early-stage EOC gradually increased (**Figure 1C**), which may result from the progress of surgical procedure-level, the improvement of medical conditions and the changing philosophy of lymph node dissection in the early-stage EOC (25).

**Figure 2:**
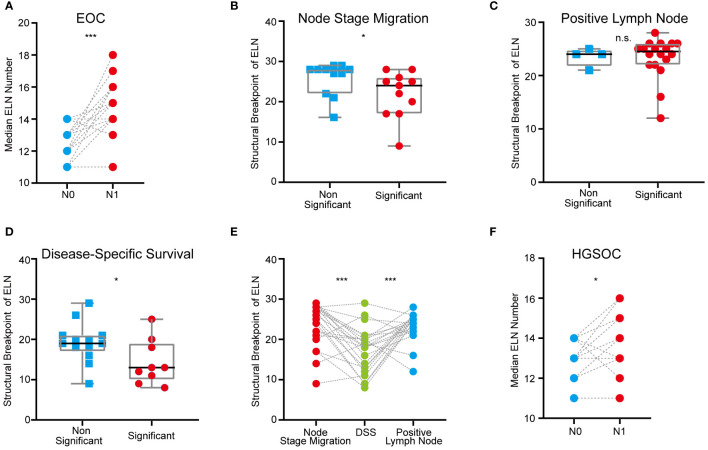
ELN numbers for node stage migration, PLN and DSS in different clinical-pathological subgroups of early-stage EOC. **(A)** Dot plot comparing median ELN numbers for stage N0 versus N1 patients in different subgroups of overall early-stage EOC. **(B)** Boxplot of ELN structural breakpoints for node stage migration in non-significant and significant subgroups from **Table 2**. **(C)** Boxplot of ELN structural breakpoints for PLN in non-significant and significant subgroups from **Table 2**. **(D)** Boxplot of ELN structural breakpoints for DSS in non-significant and significant subgroups from **Table 2**. **(E)** Dot plot comparing ELN structural breakpoints for node stage migration, PLN and DSS in different subgroups. **(F)** Dot plot comparing median ELN numbers for stage N0 versus N1 patients in different subgroups of early-stage HGSOC. P values presented in **(A)**, **(E)** and **(F)** are paired t tests. P values presented in **(B–D)** are Mann-Whitney test calculations. P values: *p < 0.05, ***p < 0.001, n.s. not significantly different.

Among the different clinical-pathological subgroups (**Table S1**), proportions of stage N1 patients ranged from 1.8% to 11.9% and increased with age. Expectedly, patients with stage T1b and T1c, bilateral laterality, grade 3 & 4 or serous disease were more likely to be stage N1 than others. Compared with the cases originated from ovary, the percentage of lymph node metastasis was higher in EOC originated from fallopian tubes. Furthermore, we found that stage N1 cases commonly had more ELN numbers than stage N0 cases (p < 0.001, **Figure 2A**), which suggested that detecting latent or occult PLN may require more ELN numbers.

### 3.2 The optimal ELN number for overall early-stage EOC

**Figure 3:**
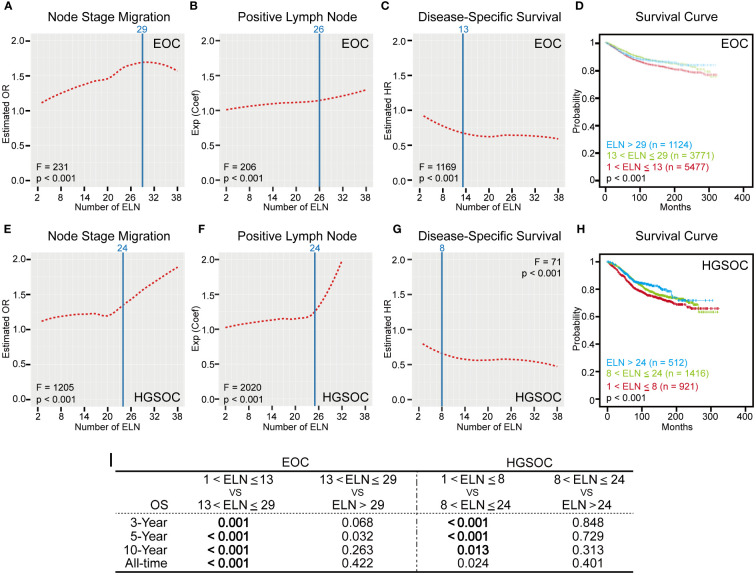
Numbers of ELN for overall early-stage EOC and HGSOC. **(A, E)** LOESS fitting curves based on ORs in ascending order of the number of ELN for early-stage EOC and HGSOC. **(B, F)** LOESS fitting curves based on exp (Coef) in ascending order of the number of ELN for early-stage EOC and HGSOC. **(C, G)** LOESS fitting curves based on HRs in ascending order of the number of ELN for early-stage EOC and HGSOC. ELN structural breakpoints for node stage migration, PLN and DSS based on Chow test are shown as blue lines in **(A–C)** and **(E–G)**. **(D, H)** Kaplan-Meier plots for DSS based on ELN structural breakpoints of early-stage EOC and HGSOC. **(I)** Log-rank test P values for DSS of different ELN subgroups. P values less than 0.016 are statistically significant.

In order to determine the ELN number for early-stage EOC, we first observed dynamic trends of associations between ELN numbers and lymph node stage migration, PLN and patient outcomes by three different regression models. When analyzed as continuous variables (**Table 1**), more ELNs were correlated with increased odds for negative-to-positive node stage migration (Logistic OR: 1.015, 95% CI: 1.008~1.022, p < 0.001), more PLNs (Linear coefficient: 0.009, 95% CI: 0.007~0.011, p < 0.001) and decreased hazard for survival (Cox HR: 0.989, 95% CI: 0.984~0.994, p < 0.001). When different ELNs were analyzed as dichotomous variables, we arranged OR, exp (Coef) and HR values of different ELNs in ascending order of the number of ELN, followed by LOESS regression fit (**Figures 3A–C**). Fitted smooth curves indicated that the probability of finding lymph node metastasis, the detectable PLN numbers and the survival gradually increased as the ELN number increased. Regardless of regression models and variable types, trends of these associations were highly consistent and independent of factors such as age, TNM stage, laterality, primary site, differentiation and histopathological subtype.

**Table 1:** Associations of ELN (as a continuous variable) with Stage Migration, PLN and DSS in different subgroups and regression models.

	Node Stage Migration (Logistic^a^)			Positive Lymph Node (Linear^b^)			Disease-Specific Survival (Cox^c^)		
Subgroup	OR	95% CI	*P*_OR_	Coef	95% CI	*P*_coef_	HR	95% CI	*P*_HR_
**Total**	1.015	1.008~1.022	**< 0.001**	0.009	0.007~0.011	**< 0.001**	0.989	0.984~0.994	**< 0.001**
Age									
≤ 50	1.011	0.998~1.023	0.099	0.011	0.007~0.014	**< 0.001**	0.994	0.985~1.003	0.227
50 ~ 60	1.016	1.004~1.028	**0.011**	0.005	0.002~0.007	**< 0.001**	0.988	0.979~0.997	**0.008**
60 ~ 70	1.023	1.008~1.038	**0.002**	0.014	0.008~0.019	**< 0.001**	0.991	0.981~1.002	0.109
> 70	1.006	0.986~1.026	0.551	0.007	0.003~0.010	**< 0.001**	0.980	0.966~0.995	**0.007**
T stage									
T1a	1.016	1.005~1.027	**0.004**	0.008	0.005~0.010	**< 0.001**	0.988	0.981~0.996	**0.004**
T1b	1.019	0.998~1.039	0.072	0.026	0.018~0.035	**< 0.001**	0.990	0.973~1.009	0.297
T1c	1.013	1.003~1.024	**0.009**	0.009	0.006~0.012	**< 0.001**	0.989	0.982~0.996	**0.004**
N stage									
N0							0.991	0.985~0.996	**0.001**
N1				0.125	0.096~0.153	**< 0.001**	0.983	0.970~0.996	**0.011**
Laterality									
Left	1.009	0.997~1.021	0.159	0.002	0.001~0.003	**< 0.001**	0.989	0.981~0.997	**0.005**
Right	1.016	1.004~1.027	**0.006**	0.008	0.005~0.010	**< 0.001**	0.989	0.981~0.997	**0.008**
Bilateral	1.022	1.008~1.036	**0.002**	0.039	0.027~0.050	**< 0.001**	0.990	0.979~1.001	0.084
Primary Site									
Ovary	1.014	1.007~1.022	**< 0.001**	0.007	0.005~0.008	**< 0.001**	0.989	0.984~0.994	**< 0.001**
Fallopian tube	1.022	0.998~1.046	0.078	0.065	0.042~0.088	**< 0.001**	0.996	0.972~1.022	0.779
Differentiation									
Grade 1	1.016	0.994~1.039	0.145	0.000	0.000~0.001	0.110	0.991	0.978~1.005	0.216
Grade 2	1.027	1.012~1.042	**< 0.001**	0.012	0.008~0.016	**< 0.001**	0.994	0.984~1.003	0.198
Grade 3&4	1.011	1.003~1.020	**0.008**	0.012	0.009~0.016	**< 0.001**	0.987	0.981~0.994	**< 0.001**
Histopathology									
Serous	1.013	1.004~1.023	**0.006**	0.016	0.011~0.020	**< 0.001**	0.989	0.981~0.997	**0.006**
Endometrioid	1.014	0.994~1.034	0.168	0.000	0.000~0.001	0.206	0.991	0.978~1.003	0.146
Mucinous	1.021	0.991~1.052	0.172	0.003	0.002~0.005	**< 0.001**	0.997	0.982~1.012	0.680
Clear cell	1.005	0.984~1.027	0.628	0.000	-0.001~0.002	0.654	0.987	0.974~1.001	0.067
Carcinosarcoma	1.021	0.939~1.110	0.634	0.018	0.000~0.036	**0.045**	0.984	0.939~1.031	0.501
Brenner & NOS	1.033	1.009~1.057	**0.006**	0.073	0.048~0.098	**< 0.001**	0.992	0.975~1.009	0.367
Mix	1.018	0.990~1.046	0.205	0.002	-0.001~0.006	0.175	0.982	0.963~1.001	0.061

**^a^** A linear relationship existed between ELN and logit N stage, variance inflation factors between independent variables were less than 10.

**^b^** Residuals with uniform variances were approximately normally distributed and had no autocorrelation. Correlation coefficients between independent variables were less than 0.7 and variance inflation factors were less than 10.

**^c^** Covariates violating the proportional hazards assumption were added as time-dependent covariates in the Cox regression models.

All p values less than 0.05 with statistical significance were use bold fronts.

However, in reality, the number of ELN cannot increase indefinitely during the surgical procedures. In addition, when the ELN number increased to a certain extent, the effect of avoiding stage migration and improving outcomes would reach the limit and saturation, which were shown as the vertex or plateaus of LOESS fitting curves (**Figures 3A, C**). In other words, too many ELNs exceeding a particular number may be meaningless.

Therefore, we conducted the Chow test on different fitted smooth curves to determine the threshold OR, exp (Coef), and HR and the corresponding structural breakpoint of ELN number. For avoiding node stage migration, the maximum ELN structural breakpoint for the threshold value of estimated OR was 29 (F = 231, p < 0.001, **Figure 3A**). For detection of more PLNs, the corresponding ELN number for the threshold value of exp (Coef) was 26 (F = 206, p < 0.001, **Figure 3B**). For improvement of outcomes, the maximum ELN structural breakpoint for the threshold value of estimated HR was 13 (F = 1169, p < 0.001, **Figure 3C**). These cut-off numbers of ELN suggested that, compared with improving outcomes, finding occult or more PLNs may require more ELNs. But what we don’t know for sure is whether more and more ELNs lead to better and better prognosis. To address this, we plotted survival curves for different early-stage EOC subgroups using ELN 13 and 29 as cut-off values (**Figure 3D**). Although patients with ELN ≤ 13 had the worst outcomes, the DSS of patient with ELN > 29 were similar to those of patients with ELN between 13 and 29 (Log-rank test p > 0.016, **Figure 3I**), which fitted well with the trend suggested by the LOESS curve.

### 3.3 ELN numbers for different clinical-pathological subgroups of early-stage EOC

Exploratory subgroup analyses are warranted due to different clinical-pathological features resulting in different risk of lymph node metastasis and mortality. As a continuous variable, ELNs were independent risk factors for lymph node stage migration in subgroups of age 50~70, stage T1a and T1c, right and bilateral laterality, ovary-origin, high grade and serous EOC (**Table 1**). These subgroups generally had smaller ELN structural breakpoints than subgroups in which ELNs were not independent risk factors for stage migration (p = 0.040, **Table 2** and **Figure 2B**). In the case of linear regression with PLNs as outcome variables, the number of ELN were meaningless only in subgroups of grade 1, endometrioid, clear cell and mix EOC (**Table 1**), whose ELN structural breakpoints were similar to other subgroups (p = 0.554, **Table 2** and **Figure 2C**). In terms of subgroup analysis by Cox regression, ELNs were independent risk factors for DSS in subgroups of age 50~60 and > 70, stage T1a and T1c, stage N0 and N1, right and left laterality, ovary-origin, high grade and serous EOC (**Table 1**). Structural breakpoints of ELN number for these subgroups were commonly less than those for subgroups where ELN were not independent risk factors for DSS (p = 0.038, **Table 2** and **Figure 2D**). By comparing ELN structural breakpoints of different regression models in the same subgroup, we found that more ELN numbers were indeed required for detecting occult or more PLNs compared with those for improving outcomes of early-stage EOC (p < 0.001, **Table 2** and **Figure 2E**).

**Table 2:** Structural breakpoints of ELN number based on ORs, Coefficients and HRs in different subgroups.

Subgroup	Node Stage Migration			Positive Lymph Node			Disease-Specific Survival		
	Structural Breakpoint	*F* ^a^	*P* ^b^	Structural Breakpoint	*F* ^a^	*P* ^b^	Structural Breakpoint	*F* ^a^	*P* ^b^
Age									
≤ 50	28	409	**< 0.001**	**22**	2123	**< 0.001**	18	9591	**< 0.001**
50 ~ 60	**9**	902	**< 0.001**	**25**	1706	**< 0.001**	**11**	1102	**< 0.001**
60 ~ 70	**20**	2801	**< 0.001**	**21**	1033	**< 0.001**	26	231	**< 0.001**
> 70	21	348	**< 0.001**	**12**	1683	**< 0.001**	**20**	104	**< 0.001**
T stage									
T1a	**26**	1792	**< 0.001**	**25**	1113	**< 0.001**	**18**	2222	**< 0.001**
T1b	28	166	**< 0.001**	**26**	1882	**< 0.001**	29	76	**< 0.001**
T1c	**17**	3431	**< 0.001**	**24**	1592	**< 0.001**	**8**	145	**< 0.001**
Laterality									
Left	27	693	**< 0.001**	**26**	476	**< 0.001**	**12**	222	**< 0.001**
Right	**28**	787	**< 0.001**	**24**	730	**< 0.001**	**25**	470	**< 0.001**
Bilateral	**24**	1904	**< 0.001**	**24**	1924	**< 0.001**	9	105	**< 0.001**
Primary Site									
Ovary	**25**	78	**< 0.001**	**25**	931	**< 0.001**	**13**	1292	**< 0.001**
Fallopian tube	28	497	**< 0.001**	**22**	1064	**< 0.001**	21	47	**< 0.001**
Differentiation									
Grade 1	27	1031	**< 0.001**	24	366	**< 0.001**	21	126	**< 0.001**
Grade 2	**28**	792	**< 0.001**	**26**	964	**< 0.001**	14	1056	**< 0.001**
Grade 3&4	**17**	906	**< 0.001**	**26**	890	**< 0.001**	**13**	1264	**< 0.001**
Histopathology									
Serous	**25**	686	**< 0.001**	**25**	1952	**< 0.001**	**9**	124	**< 0.001**
Endometrioid	22	90	**< 0.001**	25	428	**< 0.001**	20	57	**< 0.001**
Mucinous	28	151	**< 0.001**	**28**	124	**< 0.001**	20	132	**< 0.001**
Clear cell	29	194	**< 0.001**	21	46	**< 0.001**	16	1329	**< 0.001**
Carcinosarcoma	16	1537	**< 0.001**	**16**	140	**< 0.001**	19	1955	**< 0.001**
Brenner & NOS	**22**	1093	**< 0.001**	**23**	1148	**< 0.001**	18	2512	**< 0.001**
Mix	29	240	**< 0.001**	24	1029	**< 0.001**	19	726	**< 0.001**

**^a^** The F-test for the Chow Test at the given structural breakpoint.

**^b^** The P value was for the Chow Test (F test) at the given structural breakpoint.

All p values less than 0.05 with statistical significance were use bold fronts.

### 3.4 The minimal ELN number for early-stage HGSOC

As the above analysis suggested that the ELN number required may not be exactly the same for different EOC subgroups (**Table 2**), we next focused on the EOC subtype with the highest incidence, the highest degree of malignancy and the highest mortality rate, namely HGSOC (1, 2). Similar to the overall early-stage EOC, the median number of ELN and PLN was 13 and 2 (**Table S2**), 95% of early-stage HGSOC had less than 38 ELNs, 95% of stage N1 HGSOC had less than 11 PLNs (**Figures 1A, B**) and the ELN number gradually increased by the year of diagnosis (**Figure 1C**). Older early-stage HGSOC and patients with stage T1b and T1c, bilateral laterality, grade 3 & 4 or fallopian-origin disease were more likely to be stage N1 than others (**Table S2**). Stage N1 HGSOC commonly had more ELN numbers than stage N0 cases (p = 0.021, **Figure 2F**).

Likewise, as continuous variables in early-stage HGSOC (**Table 3**), more ELNs were correlated with increased odds for negative-to-positive node stage migration (Logistic OR: 1.014, 95% CI: 1.004~1.024, p = 0.007), more PLNs (Linear coefficient: 0.017, 95% CI: 0.012~0.022, p < 0.001) and decreased hazard for survival (Cox HR: 0.990, 95% CI: 0.981~0.998, p = 0.012). When different ELNs were dichotomous variables, the ORs, exp (Coef) and HRs of different ELNs were arranged in ascending order of the number of ELN and fitted by LOESS regression, which shown that the probability of detecting lymph node metastasis, the detectable PLN numbers and the survival rate gradually increased as the number of ELN increased (**Figures 3E–G**).

**Table 3:** Associations of ELN (as a continuous variable) with Stage Migration, PLN and DSS in HGSOC with stage T1M0.

	Node Stage Migration (Logistic^a^)			Positive Lymph Node (Linear^b^)			Disease-Specific Survival (Cox^c^)		
	OR	95% CI	*P*_OR_	Coef	95% CI	*P*_coef_	HR	95% CI	*P*_HR_
**ELN**	1.014	1.004~1.024	**0.007**	0.017	0.012~0.022	**< 0.001**	0.990	0.981~0.998	**0.012**
**Age**			0.010	-0.027	-0.083~0.028	0.330			< 0.001
≤ 50	1						1		
50 ~ 60	0.520	0.355~0.763	0.001				0.407	0.314~0.527	< 0.001
60 ~ 70	0.707	0.505~0.989	0.043				0.560	0.446~0.702	< 0.001
> 70	0.691	0.481~0.992	0.045				0.660	0.522~0.835	0.001
**T stage**			0.097	0.008	-0.054~0.070	0.798			< 0.001
T1a	1						1		
T1b	0.747	0.556~1.002	0.052				0.675	0.557~0.819	< 0.001
T1c	1.164	0.786~1.723	0.448				0.642	0.468~0.879	0.006
**N stage**				3.239	3.051~3.426	< 0.001			< 0.001
N0							1		
N1							0.515	0.413~0.643	
**Laterality**			< 0.001	0.036	-0.043~0.114	0.372			0.391
Left	1						1		
Right	0.436	0.300~0.633	< 0.001				0.835	0.650~1.073	0.158
Bilateral	0.623	0.437~0.889	0.009				0.842	0.658~1.077	0.171
**Primary Site**			0.006	0.308	0.122~0.495	0.001			0.536
Ovary	1						1		
Fallopian tube	0.599	0.415~0.862					1.100	0.813~1.490	
**Differentiation**			0.044	-0.005	-0.147~0.138	0.948			< 0.001
Grade 2	1						1		
Grade 3&4	0.702	0.497~0.991					0.662	0.528~0.830	

**^a^** A linear relationship existed between ELN and logit N stage, variance inflation factors between independent variables were less than 10.

**^b^** Residuals with uniform variances were approximately normally distributed and had no autocorrelation. Correlation coefficients between independent variables were less than 0.7 and variance inflation factors were less than 10.

**^c^** Covariates violating the proportional hazards assumption were added as time-dependent covariates in the Cox regression models.

All p values less than 0.05 with statistical significance were use bold fronts.

However, it is important to note that dynamic trends of associations between ELN numbers and lymph node stage migration (**Figure 3E**) and PLN (**Figure 3F**) in early-stage HGSOC were not wholly identical with those in the overall early-stage EOC (**Figures 3A, B**). The second half of LOESS curves in **Figures 3E, F** had steep rises, which means that, if ELNs exceed a particular number, the effect of avoiding stage migration and increasing the number of PLN could be substantially improved. In other words, this specific number of ELN is the minimal ELN number for early-stage HGSOC to find occult or more PLNs.

Once again, we used the Chow test on different fitted smooth curves to determine the threshold OR, exp (Coef), and HR and the corresponding structural breakpoint of ELN number. For both avoiding node stage migration and detection of more PLNs, the minimal ELN number for the threshold value of estimated OR and exp (Coef) was 24 (Node stage migration: F = 1205, p < 0.001, **Figure 3E**; PLN: F = 2020, p < 0.001, **Figure 3F**). For improvement of outcomes, the corresponding ELN number for the threshold value of estimated HR was 8 (F = 71, p < 0.001, **Figure 3G**). Finally, we plotted survival curves for different early-stage HGSOC subgroups using ELN 8 and 24 as cut-off values (**Figure 3H**). Although patients with ELN ≤ 8 had the worst DSS, outcomes of patient with ELN > 24 were similar to those of patient with ELN between 8 and 24 (Log-rank test p > 0.016, **Figure 3I**).

## 4 Discussion

In this study, generalized linear model with multivariable adjustment suggested that more ELNs were associated with more observed node-positive diseases, more PLNs and better prognosis. By the analysis strategy combining LOESS regression and Chow test, we found that different subgroups of early-stage EOC had different suitable ELN numbers and ELN numbers for detecting occult or more PLNs were larger than those for improving outcomes. The optimal number of ELN for early-stage EOC to avoid node stage migration and achieve better prognosis was 29 and 13 respectively, which was 24 and 8 for early-stage HGSOC. Survival analysis based on above results showed that the number of ELN for node stage migration were partially correlated with much better prognosis.

Since the primary lesions of early-stage EOC are limited in scope, bilateral salpingo-oophorectomy and hysterectomy provide a greater possibility of achieving R0 resection theoretically and practically, which means that most patients with early-stage EOC can achieve complete clinical cure of cancers through surgical treatment. In reality, however, this is not absolute. 10% ~ 35% early-stage EOC will relapse or die within 5 years (1, 2). Occult disease from the retroperitoneum may play an important role in it, and may be the major lesion resulting in stage migration. With the satisfactory lymphadenectomy, surgeons have a greater probability of discovering relatively occult tumor-infiltrated lymph nodes and discovering the false early-stage disease. Several prospective studies using systematic lymphadenectomy have found 3% ~ 14% of appearing early-stage patients had PLNs (9, 10, 26–29). When we accurately distinguish early-stage and advanced EOC, the occurrence of stage migration can be effectively reduced, which can lead to significantly superior long-term prognosis. Thus, lymph node stage migration should be an important factor in determining the number of ELN for the surgery of early-stage EOC (21, 22, 30–32). In clinical work, FDG-PET/CT may be the most sensitive examination for lymph node stage migration, but has unclear significance for early-stage EOC (33, 34). So far, recommendations on ELN numbers for early-stage EOC have not been uniform and reasonable (12–17), and to our knowledge, this current study is the only one aimed at detecting occult or potential PLN to avoid node stage migration and proposing well-founded numbers of ELN according to assumptions of appropriate statistical tests.

When different ELNs are dichotomous variables, it is easy to know ORs for node stage migration, coefficients for PLN and HRs for survival. In general, the larger the number of ELNs, the larger the OR and the coefficient and the smaller the HR. So it would be highly impractical to determine the suitable number of ELN based on the maximum values of OR and coefficient and the minimum value of HR. Instead, it may be a more reasonable strategy to choose the ELN number by reference to the inflection point based on the continuous and dynamic change of LOESS curves of OR, coefficient and HR. In our study, ELN numbers corresponding to two kinds of inflection point location were identified by the Chow test. The number of ELN corresponding to either the vertex (**Figure 3A**) or the starting point for the plateau of fitted curves (**Figures 3C, G**) may be the maximum ELN number for surgery, since too many ELNs provided no more benefits. The number of ELN corresponding to the end point for the plateau of fitted curves (**Figures 3B, E, F**) may be the minimum number of ELN for surgery, since more ELNs provided more benefits. The numbers of ELN used for node stage migration and PLN were similar or even identical, which indirectly reflects the stability and reliability of our analysis strategy combining LOESS regression and Chow test. Moreover, the NCCN Guidelines recommend 6 cycles of adjuvant chemotherapy for stage I HGSOC and 3 cycles for other stage I EOC. More cycles of chemotherapy for early-stage HGSOC may partially alleviate the effect of stage migration. Instead, of early-stage patients with enough ELNs, there was no difference in relative survival between those who received chemotherapy and those who did not (13). Our finding that the minimum ELN number for early-stage HGSOC should be clear to avoid stage migration as much as possible was consistent with the notion that more ELNs and chemotherapy make up for the negative prognostic impact of the highly malignant biological behavior of HGSOC.

Although more ELNs were associated with higher survival rate in multivariable-adjusted Cox models of both early-stage EOC and extensive stratifications (**Tables 1** and **3**), it does not mean that survival rates can be infinitely improved by increasing the numbers of ELN. LOESS curves and Chow test based on HRs of different ELNs shown that the effect of improving outcomes have already reached the limit and saturation at small ELN numbers (**Table 2**, **Figures 2E**, **3C, G**). Our survival analyses and other previous studies also have come to a similar conclusion that too many ELNs were meaningless for the improvement of prognosis (**Figures 3G, H, I**) (9, 11–13, 19). For patients with presumed early stage, a randomized trial showed that systematic aortic and pelvic lymphadenectomy was not associated with improved progression free survival or overall survival (9). However, meta-analyses that included retrospective or observational studies have reported that systematic lymphadenectomy improves overall survival in patients with early-stage disease, even though it does not improve progression free survival (35, 36). Considering that it remains uncertain whether the difference in prognosis resulting from different numbers of ELN is concealed by conventional postoperative adjuvant chemotherapy (13), namely whether the benefit of detecting stage migration could be reflected directly and completely by better prognosis is still unknown, the evaluation of the number of ELN only from the perspective of prognosis alone may not be comprehensive and objective. Thus, for surgery, we would prefer the numbers of ELN based on node stage migration and PLN.

Although current guidelines still recommend laparotomy for early-stage ovarian cancer, a significant proportion of early-stage ovarian cancer is discovered incidentally during minimally invasive surgery for benign conditions of pelvic cavity. The greatest advantage of minimally invasive surgery is that it reduces intraoperative and postoperative complications and shortens the length of stay in hospital while has a similar surgery scope and effect to open surgery (37–39), which is consistent with our original intention to determine the optimal number of ELNs, that is, to remove as many lymph nodes with occult metastasis as possible while minimizing the surgical trauma by controlling the number of ELNs. Lymphadenectomy in the process of minimally invasive surgery may have the following characteristics: First, the local magnification of lymph nodes by laparoscopic and robotic surgical equipment allows the surgeon to obtain a clearer view of the surgical field during lymphadenectomy; Secondly, median numbers of ELNs in minimally invasive surgery and laparotomy were similar, and there was no significant reduction (40–43). Third, minimally invasive surgery is easier to perform sentinel lymph node biopsy than open surgery. Given that a large number of retrospective studies and meta-analyses suggested that the oncologic outcome in patients with minimally invasive surgery was no worse than that in patients undergoing open surgery (a reliable conclusion even with prolonged follow-up) (37, 39, 41–44), we believe that lymphadenectomy by minimally invasive surgery may be beneficial for early-stage ovarian cancer.

Another way to detect occult metastatic lymph nodes as much as possible while reducing surgical trauma is sentinel lymph node resection or biopsy (5–7, 45). However, considering that pure sentinel lymph node resection may only have 6~7 ELNs (46–50), there is still a great risk of missing diagnosis. So targeted compartmental lymphadenectomy, a modified lymphadenectomy that removes embryologically defined compartments of locoregional tumor spread with the help of sentinel lymph node mapping, was proposed (4–7). It reduces perioperative complications compared with systematic lymph node dissection. At the same time, compared with traditional sentinel lymph node biopsy, it enhances the safety and reliability of diagnosis (51–53). More importantly, compartmental surgery has been shown to be effective in controlling locoregional tumor recurrence in retroperitoneal sarcomas and mouth cancers (54–58), that is, improving oncologic outcomes of patients. Our study is only a retrospective description of the number of ELNs. It is impractical and undesirable to perform lymphadenectomy solely based on the number of ELNs and to ignore the different metastatic risks of lymph nodes with different locations and spatial distributions. The targeted compartmental lymphadenectomy provides us with information on the different locations and spatial distribution of lymph nodes, and our research conclusion can also provide the targeted compartmental lymphadenectomy with a reference in the number of ELNs, partially and indirectly. There is a potential for reasonable joint application of the two, which may replace the traditional sentinel lymphadenectomy and the random or systemic lymphadenectomy.

In spite of a certain degree of rationality and robustness, our study has certain limitations. First, we only discuss associations between the number of ELN and stage migration, PLN and DSS, but do not know the spatial distribution of ELNs. It is difficult to estimate the impact of draining lymph nodes with different anatomic locations, such as iliac vascular lymph nodes or para-aortic lymph nodes, on different subgroups of early-stage EOC (10). Second, in the course of clinical practice, other confounders, including but not limited to BMI, anatomic variants, tumor heterogeneity, comorbidities, complications and pre-operative therapy, were not available in our study, but may also substantially influences the number of ELN. Third, as a retrospective descriptive study of the number of ELNs, our conclusions cannot be used as a guide or recommendation for lymphadenectomy. In the future, we look forward to further retrospective and prospective studies based on any other databases or hospitals to validate our ELN structural breakpoints.

In conclusion, for early-stage EOC and HGSOC, neither too many nor too few numbers of ELN were ideal and we do not encourage traditional sentinel lymph node resection or systematic lymphadenectomy. Excision with appropriate numbers of lymph node draining the affected ovary may be a reasonable choice.

## Data availability statement

Publicly available datasets were analyzed in this study. This data can be found here: https://seer.cancer.gov/data-software/documentation/seerstat/.

## Author contributions

YL: Formal Analysis, Funding acquisition, Investigation, Writing – original draft, Writing – review & editing. JD: Formal Analysis, Investigation, Software, Writing – original draft, Writing – review & editing, Formal Analysis, Investigation, Software, Writing – original draft, Writing – review & editing. HZ: Methodology, Writing – original draft. LX: Data curation, Writing – original draft. WL: Funding acquisition, Writing – original draft. MZ: Data curation, Writing – original draft. XZ: Data curation, Writing – original draft. CM: Supervision, Writing – original draft. FZ: Supervision, Writing – original draft. PZ: Visualization, Writing – original draft. DL: Visualization, Writing – original draft. YH: Visualization, Writing – original draft. SZ: Project administration, Writing – original draft, Writing – review & editing. LH: Funding acquisition, Writing – original draft. JL: Conceptualization, Methodology, Writing – original draft, Writing – review & editing.
