# Supplementary material for: Speculation on optimal numbers of examined lymph node for early-stage epithelial ovarian cancer from the perspective of stage migration
Source: Front Oncol. 2023 Sep 22;13:1265631. doi: 10.3389/fonc.2023.1265631 (PMC10556677; doi:10.3389/fonc.2023.1265631)
Supplement: Supplementary file 1 [file Table_1.docx]

**Table S1.** Distribution of patient number, ELN and PLN in clinical-pathological subgroups of EOC with stage T1M0.

| **Subgroup** | **Number** ^a^ | **N0 Number** ^a^ | **N0 ELN** ^b^ | **N1 number ­­**^a^ | **N1 ELN** ^b^ | **N1 PLN** ^b^ |
| --- | --- | --- | --- | --- | --- | --- |
| **Total** | 10372 (100%) | 9812 (94.6%) | 13 (2~90) | 560 (5.4%) | 14 (2~90) | 2 (1~70) |
| **Age** |  |  |  |  |  |  |
| < 50 | 3763 (100%) | 3596 (95.6%) | 13 (2~83) | 167 (4.4%) | 15 (2~90) | 1 (1~70) |
| 50 ~ 60 | 3384 (100%) | 3216 (95.0%) | 13 (2~90) | 168 (5.0%) | 15 (2~70) | 2 (1~31) |
| 60 ~ 70 | 1975 (100%) | 1845 (93.4%) | 13 (2~76) | 130 (6.6%) | 16 (2~81) | 2 (1~54) |
| > 70 | 1250 (100%) | 1155 (92.4%) | 11 (2~76) | 95 (7.6%) | 11 (2~90) | 2 (1~12) |
| **T stage** |  |  |  |  |  |  |
| T1a | 5766 (100%) | 5550 (96.3%) | 12 (2~90) | 216 (3.7%) | 15 (2~77) | 2 (1~70) |
| T1b | 624 (100%) | 551 (88.3%) | 14 (2~68) | **73 (11.7%)** | 13 (2~70) | 2 (1~18) |
| T1c | 3982 (100%) | 3711 (93.25) | 13 (2~90) | **271 (6.8%)** | 14 (2~90) | 2 (1~54) |
| **N stage** |  |  |  |  |  |  |
| N0 | 9812 (100%) | 9812 (100%) | 13 (2~90) |  |  |  |
| N1 | 560 (100%) |  |  | 560 (100%) | 14 (2~90) | 2 (1~70) |
| **Laterality** |  |  |  |  |  |  |
| Left | 4676 (100%) | 4497 (96.2%) | 13 (2~90) | 179 (3.8%) | 14 (2~90) | 2 (1~20) |
| Right | 4498 (100%) | 4259 (94.7%) | 12 (2~84) | 239 (5.3%) | 14 (2~61) | 2 (1~54) |
| Bilateral | 1198 (100%) | 1056 (88.1%) | 14 (2~73) | **142 (11.9%)** | 15 (2~90) | 2 (1~70) |
| **Primary Site** |  |  |  |  |  |  |
| Ovary | 9914 (100%) | 9410 (94.9%) | 13 (2~90) | 504 (5.1%) | 14 (2~90) | 2 (1~70) |
| Fallopian tube | 458 (100%) | 402 (87.8%) | 14 (2~60) | **56 (12.2%)** | 14 (3~62) | 2 (1~54) |
| **Differentiation** |  |  |  |  |  |  |
| Grade 1 | 2858 (100%) | 2804 (98.1%) | 12 (2~83) | 54 (1.9%) | 15 (2~48) | 1 (1~6) |
| Grade 2 | 3282 (100%) | 3179 (96.9%) | 12 (2~80) | 103 (3.1%) | 16 (2~90) | 1 (1~70) |
| Grade 3&4 | 4232 (100%) | 3829 (90.5%) | 13 (2~90) | **403 (9.5%)** | 14 (2~90) | 2 (1~54) |
| **Histopathology** |  |  |  |  |  |  |
| Serous | 3132 (100%) | 2822 (90.1%) | 13 (2~90) | **320 (10.2%)** | 14 (2~81) | 2 (1~54) |
| Endometrioid | 2953 (100%) | 2896 (98.1%) | 13 (2~80) | 56 (1.9%) | 17 (2~90) | 1 (1~6) |
| Mucinous | 1712 (100%) | 1682 (98.2%) | 11 (2~83) | **30 (1.8%)** | 16 (3~61) | 2 (1~16) |
| Clear cell | 1264 (100%) | 1198 (94.8%) | 13 (2~77) | 66 (5.2%) | 14 (2~44) | 1 (1~9) |
| Carcinosarcoma | 95 (100%) | 88 (92.6%) | 12 (2~53) | 7 (7.4%) | 18 (3~45) | 2 (1~11) |
| Brenner & NOS | 441 (100%) | 393 (89.1%) | 11 (2~76) | **48 (10.9%)** | 14 (2~90) | 2 (1~70) |
| Mix | 766 (100%) | 733 (95.7%) | 13 (2~67) | 33 (4.3%) | 16 (4~43) | 2 (1~10) |

**^a^** data are shown as count (%);

**^b^** data are shown as median (range).

**Table S2.** Distribution of patient number, ELN and PLN in clinical-pathological subgroups of HGSOC with stage T1M0.

| **Subgroup** | **Number** ^a^ | **N0 Number** ^a^ | **N0 ELN** ^b^ | **N1 number** ^a^ | **N1 ELN** ^b^ | **N1 PLN** ^b^ |
| --- | --- | --- | --- | --- | --- | --- |
| **HGSOC** | 2849 (100%) | 2550 (89.5%) | 13 (2~90) | 299 (10.5%) | 14 (2~81) | 2 (1~54) |
| **Age** |  |  |  |  |  |  |
| < 50 | 717 (100%) | 661 (92.2%) | 14 (2~73) | 56 (7.8%) | 13 (2~48) | 2 (1~19) |
| 50 ~ 60 | 949 (100%) | 845 (89%) | 13 (2~90) | 104 (11%) | 15 (2~70) | 2 (1~31) |
| 60 ~ 70 | 686 (100%) | 614 (89.5%) | 13 (2~71) | 72 (10.5%) | 16 (3~81) | 2 (1~54) |
| > 70 | 497 (100%) | 430 (86.5%) | 11 (2~56) | 67 (13.5%) | 11 (2~55) | 2 (1~12) |
| **T stage** |  |  |  |  |  |  |
| T1a | 1236 (100%) | 1140 (92.2%) | 12 (2~90) | 96 (7.8%) | 14 (2~54) | 2 (1~15) |
| T1b | 276 (100%) | 227 (82.2%) | 14 (2~59) | **49 (17.8%)** | 15 (2~70) | 2 (1~18) |
| T1c | 1337 (100%) | 1183 (88.5%) | 13 (2~90) | **154 (11.5%)** | 13 (2~81) | 2 (1~54) |
| **N stage** |  |  |  |  |  |  |
| N0 | 2550 (100%) | 2550 (100%) | 13 (2~90) |  |  |  |
| N1 | 299 (100%) |  |  | 299 (100%) | 14 (2~81) | 2 (1~54) |
| **Laterality** |  |  |  |  |  |  |
| Left | 1105 (100%) | 1025 (92.8%) | 14 (2~90) | 80 (7.2%) | 15 (2~81) | 2 (1~20) |
| Right | 1142 (100%) | 1025 (89.8%) | 12 (2~84) | 117 (10.2%) | 13 (2~61) | 2 (1~54) |
| Bilateral | 602 (100%) | 500 (83.1%) | 14 (2~73) | **102 (16.9%)** | 16 (2~70) | 2 (1~31) |
| **Primary Site** |  |  |  |  |  |  |
| Ovary | 2543 (100%) | 2287 (89.9%) | 13 (2~90) | 256 (10.1%) | 14 (2~35) | 2 (1~31) |
| Fallopian tube | 306 (100%) | 263 (85.9%) | 14 (2~59) | **43 (14.1%)** | 12 (3~62) | 2 (1~54) |
| **Differentiation** |  |  |  |  |  |  |
| Grade 2 | 577 (100%) | 535 (92.7%) | 12 (2~65) | 42 (7.3%) | 14 (2~62) | 1 (1~20) |
| Grade 3&4 | 2272 (100%) | 2015 (88.7%) | 13 (2~90) | **257 (11.3%)** | 14 (2~81) | 2 (1~54) |

**^a^** data are shown as count (%);

**^b^** data are shown as median (range).
